# Echocardiography and Newer Imaging Techniques in Diagnosis and Long-Term Follow-Up of Primary Heart Tumors in Children

**DOI:** 10.3390/ijerph17155471

**Published:** 2020-07-29

**Authors:** Aleksandra Morka, Joanna Kohut, Beata Radzymińska-Chruściel, Tomasz Mroczek, Marcin Gładki, Piotr Weryński, Andrzej Rudziński, Janusz Skalski, Lesław Szydłowski

**Affiliations:** 1 Department of Pediatric Cardiac Surgery, University Children’s Hospital, Faculty of Health Sciences, Jagiellonian University Medical College, 30-663 Kraków, Poland; 2 Department of Pediatric Cardiology, Faculty of Medical Sciences, Medical University of Silesia in Katowice, 40-752 Katowice, Poland; jkohut6@wp.pl (J.K.); szydlowskil@interia.pl (L.S.); 3 Clinic of Fetal Echocardiography, Medical Centre UJASTEK, 31-752 Kraków, Poland; bearad63@gmail.com; 4 Department of Pediatric Cardiac Surgery, University Children’s Hospital, Faculty of Medicine, Jagiellonian University Medical College, 30-663 Kraków, Poland; t_mroczek@hotmail.com (T.M.); janusz_skalski@poczta.onet.pl (J.S.); 5 Department of Pediatric Cardiac Surgery, University Children’s Hospital, 30-663 Kraków, Poland; marcingladki@gmail.com; 6 Department of Pediatric Cardiology, University Children’s Hospital, Faculty of Medicine, Jagiellonian University Medical College, 30-663 Kraków, Poland; werpiotr@interia.pl (P.W.); rudka@poczta.onet.pl (A.R.)

**Keywords:** primary heart tumor, rhabdomyoma, sclerosis tuberosa, children

## Abstract

Background: Primary heart tumors (PHTs) in the pediatric population are very rare and do not manifest any characteristic symptoms. Methods: A retrospective analysis of 61 cases was undertaken. Data from three centers for the years 2003–2018 were gathered. The tumors’ clinical course, location, number, hemodynamic, treatment, and follow-up were evaluated. Echocardiography was complemented with magnetic resonance imaging, computer tomography, and histopathological examination. Results: Out of 61 PHT diagnoses, 56 (91.8%) were circumstantial including all 16 (26.2%) prenatal tumors. The reasons for cardiological consultations were arrhythmia, syncopes, lowered physical performance, and murmurs. Only five patients (8.2%) were suspected of tumors based on previous symptoms of sclerosis tuberosa. Rhabdomyoma was the most frequently found PHT (60.7%). The tumors were predominantly located in the ventricles (49.1%) and intraventricular septum (14.9%) and tended to be single (70.5%). About 37.7% of patients suffered from coexistent multi-organ problems, two (3.28%) from congenital heart defects and one (1.64%) from Carney’s syndrome. Tumor resection was performed on 26 (42.7%) patients, of which 16 (61.5%) underwent total and 10 (38.5%) partial tumor resection. During the follow-up (mean 4.3 years), 54 patients (88.5%) have improved or were stable, while seven (11.5%) died. Conclusions: Primary pediatric heart tumors are diagnosed completely circumstantially, and the most common is rhabdomyoma, although arrhythmia may suggest fibroma. Diagnosis of a heart tumor in children is not synonymous with fatal prognosis, and most of them require only constant observation. Life-saving operation allows improvement, while the prognosis for malignant tumors in children is definitely unfavorable.

## 1. Introduction

Primary heart tumors (PHTs) in children are rare, and their frequency varies depending on the evaluation method used. In post-mortem examination, heart tumors are diagnosed in 0.0017–0.028% of patients who undergo autopsy, independent of the age group [1]. During prenatal examination, 0.14–0.17% fetuses are diagnosed with PHT [2,3,4], whereas the frequency of children diagnosed with PHT among those referred for cardiological consultation is 2.5/1000 [5]. Due to the wider application of echocardiographic screening during cardiological and prenatal consultations, PHTs are now detected more frequently. However, during both prenatal and postnatal periods, there may be no clinical symptoms of tumors, or they may cause outflow obstruction. Less commonly, they may be located in the atrioventricular groove and cause arrhythmias related to the creation of an accessory pathway. Of these symptoms, death is the most severe.

Heart tumors may be classified as primary, secondary, benign, or malignant. Pericardial tumors are grouped separately [1].

About 90% of pediatric tumors are benign, and the one most frequently encountered is rhabdomyoma (approximately 60–70%). Less common benign tumors in this group include teratoma, fibroma and hemangioma, which together account for 25–30% of cases, while the most common malignancies are angiosarcoma, fibrosarcoma, and lymphosarcoma [1,5,6].

In adults, 75% of tumors are benign primary tumors. Of these, 40% are myxoma. The remaining 25% are malignant. Primary malignant tumors are 10–20 times rarer than secondary malignant tumors, and these most commonly stem from metastasis or direct infiltrations. Clinical traits of tumors in children and adults depend on the tumor’s localization, size, pace of growth, and possible narrowing of the circulatory pathways [5,7].

The gold standard diagnostic test for pediatric heart tumors is echocardiography. However, it is commonly complemented with computed tomography (CT) or/and magnetic resonance imaging (MRI) scans because the latter provides information about both the tumor’s size and location and helps in the initial verification of the tumor’s type [8].

The treatment of heart tumors depends on a variety of factors, especially on secondary hemodynamic symptoms. In the case of rhabdomyoma, adopting a wait-and-watch attitude is most common because the majority of rhabdomyomas spontaneously regress in early childhood. Surgical resection is reserved for cases of heart failure due to outflow obstruction or severe arrhythmias.

The prognosis of malignant pediatric tumors is usually most unfavorable. However, they rarely occur in the age group [3,8].

The purpose of this study was to evaluate the initial symptoms, time of diagnosis, clinical course, diagnostic methods used, the treatment prescribed for various pediatric heart tumors, and the results of the follow-up.

## 2. Material and Methods

The clinical background was established. The sample comprised a group of 61 children (27 boys and 34 girls) who were diagnosed with benign or malignant PHT in utero/post-natal period (age: prenatal to 17.5 years old when diagnosed). The children were under observation at three centers: two cardiology clinics and a prenatal medical center during the period 2003–2018. Medical documentation data drawn from spreadsheets were analyzed.

Inclusion criteria: patients from prenatal stage to 18 years of age, who had cardiac abnormalities in the shape or structure of the heart walls, and with symptoms of sclerosis tuberosa and arrhythmias.

Exclusion criteria: age over 18, metastatic tumors, mediastinal tumors with heart involvement, family history of hypertrophic cardiomyopathy, and typical echocardiographic picture of hypertrophic cardiomyopathy.

Each patient had been subjected to detailed physical examination, basic laboratory tests, electrocardiography (ECG), and two-dimensional and Doppler echocardiography (2DE) for the routine estimation of the anatomy and function of the heart.

Echocardiography was the first imaging method of assessment. Echocardiographic examination was performed in two-dimensional (2D), and in recent studies, three-dimensional (3D) projection using transducers with a frequency of 3.5–7.0 MHz. In some cases, transesophageal echocardiography (TEE) was also used. Dyskinesis or hypokinesis was evaluated using color tissue doppler imaging (TDI). Probes were situated in subcostal and 4-chamber apical view, as well as in the parasternal notch. The function of the left ventricle was calculated per Simpson formula.

Echocardiographic examination complemented by MRI and/or CT scans established the primary diagnosis of the type of tumor, its location, and the number of tumors, and its effects on hemodynamics were evaluated. Later, the cardiovascular, central nervous, and other body systems were analyzed. Thereafter, the time of observations and the prescribed treatment were evaluated. In the case of surgical removal of tumor or the patient´s death, a histopathological examination of anatomical specimens established the final diagnosis. The last stage of the study was an evaluation of the follow-up results.

A retrospective study was undertaken. According to the Helsinki Convention, the study did not require the Bioethical Commission’s permission to be conducted.

## 3. Results

### 3.1. The General Characteristics of the Population

Out of a total of 61 tumors, 16 (26.2%) were diagnosed in utero. In one fetus, additionally, a pericardial effusion was present. Therefore, it was presumed that after-birth drainage had to be performed (Table 1).

In another case, an episode of paroxysmal supraventricular tachycardia (SVT) was noted. For two other fetuses, the routine examination showed heart tumors accompanied by congenital heart defects (Table 2).

The remaining members of the group experienced no other complications during the pregnancy and the first days after birth. There were no differences in results between natural-born and cesarean-section-born children. In one case of rhabdomyoma, a significant decrease in the tumor’s mass was observed during prenatal life (data not shown).

Of all 61 cases, only five (8.2%) children had been referred for suspected cardiac tumor (rhabdomyoma), while all the other members of the sample had a previous neurological diagnosis of sclerosis tuberosa (ST). All other heart tumors were diagnosed entirely by accident during a routine cardiac examination.

### 3.2. Types of Tumors

**Figure 1:**
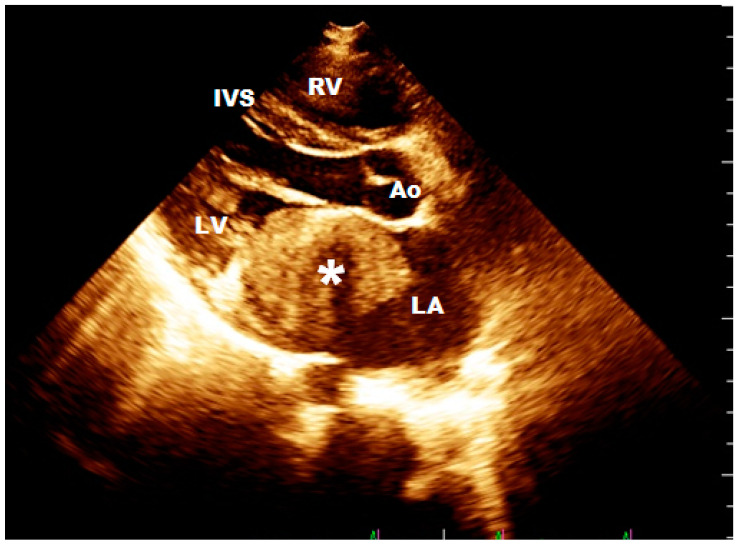
Two-dimensional echocardiography: parasternal long axis image obtained in a 16-year boy shows a huge hyperechoic mass (*) in the left atrium (LA) which was significantly hindering the flow of blood to the left ventricle through the mitral valve. RV—Right Ventricle; IVS—Interventricular Septum; LV— Left Ventricle; LA—Left Atrium; Ao—Aorta.

**Figure 2:**
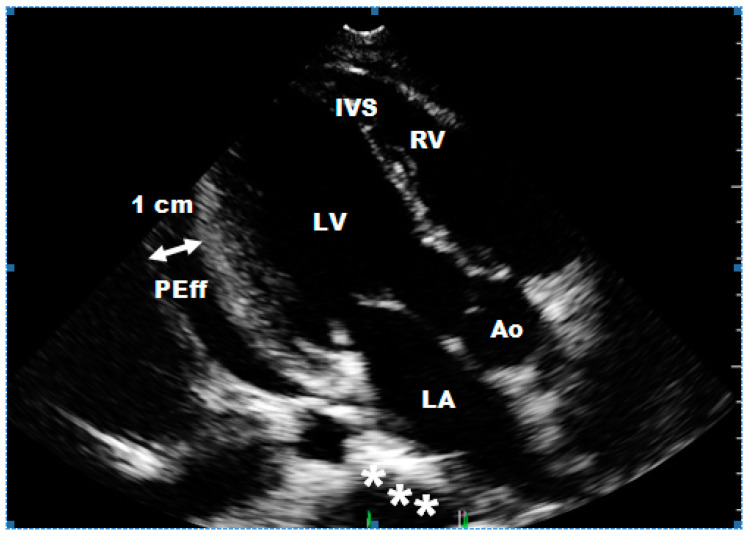
Two-dimensional echocardiography: parasternal long axis image obtained in the same patient as in Figure 1, after subtotal resection of tumor. Although the tumor was removed completely, infiltration of the left atrial wall was found. The thickening of the left atrial wall and its irregular shape are clearly visible (*). Pericardial effusion (PEff) is also present about 1 cm behind the posterior wall of the LV. Abbreviations as in Figure 1.

The most commonly diagnosed tumor was rhabdomyoma, present in 37 (60.7%) cases. The next was fibroma, 13 (21.3%), and the third most frequent was teratoma, noted in five (8.2%) cases. Myxoma was diagnosed only in three (4.9%) cases. The remaining tumors were malignant and mesothelioma and rhabdomyosarcoma (Figure 1 and Figure 2) were diagnosed incidentally (Table 3).

### 3.3. Tumor Location

In 30 (49.1%) patients, the tumors were located in the ventricles, and in nine (14.9%) cases, they were found in the interventricular septum. The remaining tumors appeared in the atria, pericardium, or at multiple locations at once (Table 4).

### 3.4. Number of Tumors

The diagnosis indicated 70.5% of tumors to be single (Table 5).

Among single tumors, ST was reported in 9.3% only. However, when two to three tumors were found, then ST occurred in 71.2%, while in more than three tumors, ST was found in 90.9%. This indicates that if multiple heart tumors are detected, it can be a rhabdomyoma.

All patients underwent basic tests such as morphology of platelets with numbers, bilirubin, urea, creatinine, and some also aminotransferase. However, they did not reveal any valuable information (data not shown).

### 3.5. Diagnostic Methods

Echocardiography, owing to which the tumors were diagnosed, was performed on all the patients (100%).

Of 33 (54%) patients who underwent chest X-ray, 11 (33%) were diagnosed with problems such as pleural fluid and enlarged heart shape, although this imaging has little diagnostic value in children with heart tumors.

Twenty-four patients were subjected to brain ultrasonography and one presented hemorrhagic alterations connected with deep fetal bradycardia and resuscitation on the first day of life. The remaining ultrasound scans were unaltered, although seven were supplemented with brain MRIs, which indicated well-circumscribed areas of high-signal intensity leading to a final diagnosis of ST.

Routine ECG was performed on all (100%) children, while 24-h Holter was used on 25 patients (40.1%). Of these, 14 (56%) showed abnormalities; nine indicated the appearance of additional supraventricular and ventricular extra systoles, while three showed SVT episodes due to pre-excitation syndrome. Furthermore, two were diagnosed with Mobitz-1 AV block and repolarization disorders. All children with fibroma had various types of cardiac arrhythmia.

Magnetic resonance (MR) was used in 16 cases and confirmed the initial diagnosis of a tumor.

In six children, the tumor’s diagnosis was confirmed with a CT scan (data not shown).

### 3.6. Treatment

Surgery was carried out in 26 (42.7%) cases. Of these, 17 (65.4%) were performed urgently and eight (34.6%) were planned surgeries. Total resection was performed in 16 (61.5%) cases and partial in 10 (38.5%). In a 17-month post-operation follow-up, an improvement in/stabilization of the current state was indicated in 22 (84.6%) cases, while four patients (approximately 15.4%) died, including all who had undergone urgent surgery for a malignant tumor (Table 3 and Table 6).

## 4. Discussion

Neonatal and pediatric PHTs differ in growth dynamics and clinical course. Careddu et al. [7] point out that the symptoms indicating fetal heart tumors are arrhythmia, congestive heart failure, and hydrops. Heart tumors are frequently diagnosed circumstantially while investigating a faulty mass of tissue during a routine obstetric examination [2]. In the population selected for this research, 75% of diagnoses of neonatal tumors were circumstantial and discovered during a routine ultrasonographic examination. The remaining 25% of newborns had shown life-threatening symptoms such as heart tamponade, supraventricular tachycardia due to Wolf-Parkinson-White syndrome, and in the case of two infants, congenital heart defects.

Our research shows that tumors in children occur with a similar frequency in both girls and boys. On the other hand, the three most common tumors in childhood (rhabdomyoma, fibroma and teratoma) are detected very early in infancy, while tumors characteristic of adults (myxomas) and malignant tumors appear above the age of 10.

The clinical systemic signs and symptoms depend on the tumor’s location, size, and its influence on hemodynamics and the conduction system and can include weight loss, malaise, fatigue, anemia and fever. In laboratory tests, there are elevated white blood cell counts and elevated blood platelet counts [8,9,10,11,12].

The other symptoms observed in heart tumors are murmur, tachypnea, cyanosis, manifestations of heart failure, and various types of arrhythmia [13,14,15]. Older patients reported a reduction in the tolerance for physical activity, as well as arrhythmia, dyspnea, fainting, and chest pain. Other symptoms, such as a significant loss of blood flow and/or severe arrhythmias, may cause sudden cardiac death. However, the most common clinical picture still consists of nonspecific symptoms [16,17].

In our study, only five children (8.2%) were suspected to have a tumor based on traits of ST confirmed by neurological diagnosis, while the remaining tumors were diagnosed circumstantially when referred for a cardiovascular check-up due to unspecified symptoms such as cardiac murmur, arrhythmia, reduced tolerance for physical activity, and syncope.

The most dangerous traits are obstructions in intracardiac blood flow and peripheral embolization. According to Yin et al. [18], blood flow-obstructing stenoses were diagnosed in 60–70% of adult patients affected by heart tumors, and stenocardial pain and loss of consciousness were the main clinical symptoms. Embolization was found in 21.5% of cases. Among patients suffering from heart tumors, especially myxoma and heart sarcoma, this may result in brain strokes or pulmonary embolism [19,20]. The occurrence of embolic complications among children with a confirmed diagnosis of heart tumor in this study was rare, and this concurred with the general finding that tumors with this complication are relatively rare in this age group.

In our research, only one patient, a girl with myxoma, suggested the possibility of an embolic-type complication (sight deficiencies, fainting) in whom we initially considered Transient Ischemic Attack (TIA).

Arrhythmias that accompany heart tumors usually occur when they infiltrate the conductive tissue. This blocks conduction and distorts the heart cycle. In addition, tumors can disrupt the electrical and contractile function of the heart, resulting in atrial/ventricular tachyarrhythmias [8].

According to Careddu et al. [7], in the case of a confirmed pediatric arrhythmia, especially a ventricular one, it is important to confirm the presence of a heart tumor. Other symptoms are the lack of weight gain, as well as anorexia, polycythemia, leukocytosis, and thrombocytosis. It is common for heart tumors to increase the levels of proinflammatory cytokines (interleukin 6, especially in myxoma).

In our material, a Holter EKG was performed in 25 children (40.1%), and 14 (56%) of them were diagnosed with various rhythm and conduction disorders. An indication for the Holter EKG was irregular heart rhythm in clinical examination and/or additional heart contractions on routine EKG.

The diagnosis of a tumor is based mainly on echocardiography. It can define the location, number, and the size of tumors and provides real-time functional information including tumor mobility as well as its influence on hemodynamics and adjoining structures. Cardiac contractility, valve incompetence, and the presence of pericardial fluid are assessed as well. Doppler echocardiography provides blood velocity data so that it is possible to assess blood flow disorders caused by a heart tumor. Three-dimensional transthoracic and transesophageal echocardiography can help locate and characterize heart cancers during preoperative planning and surgery. It is claimed that the echocardiographic image correlates well with the intraoperative picture [8].

In our material, all tumors were diagnosed from echocardiographic examinations, of which 16 (26.2%) were performed in utero. The results of the examinations correlated well with the intraoperative picture or with MRI and CT scans.

It is emphasized that echocardiography offers relatively poor visualization of soft tissue and cardiac tumor infiltration compared to CT and MR imaging. While echocardiography is sufficient to diagnose a tumor, CT and MRI currently have a leading role in diagnosing the type. So, both CT and MRI are increasingly important in diagnosing, characterizing, and planning treatment strategies for cardiac tumors [8].

Angiography is currently occasionally used to diagnose tumors, but is still a useful tool to evaluate coronary artery disease (in older adults) or to assess a tumor’s vascularity.

Though visualization methods have improved continuously, the most vital part of a successful treatment plan and assessment of prognosis is a careful and precise histopathological analysis of biopsy material [7,21].

In our research, the most common tumor was rhabdomyoma. This kind of tumor may be singular or plural, mostly located in the myocardium. Partial/complete regression is observed in approximately 54% [4,22,23] of cases. We concluded from our own observations that even though some of the tumors spontaneously decreased in size or even regressed, the neurological symptoms (epilepsy) can increase. It was observed in this study that 19 children (31.1%) suffered from ST, of which five were referred by the neurologist for a cardiological check-up with “heart tumor” pre-diagnosis, which was later confirmed. On the other hand, in a group of 12 patients (19.6%) with rhabdomyoma, who had electroencephalogram in six (50%), the result was abnormal and ST symptoms were developed. Therefore, it is so important to provide complex observations, diagnostics, and treatment plans for patients with tumors at the prenatal stages [11,24,25,26,27]. Rhabdomyoma is usually located in the ventricular myocardium. In our study, it appeared in 37 cases (60.7%), and this occurrence was similar to what other authors suggest [4,7,9,13,17]. Black et al. [22] stated that out of 30 children with rhabdomyoma, 23 (77%) showed spontaneous regression. Therefore, due to high potential of spontaneous regression, a surgical intervention is needed only if the tumor causes significant hemodynamic disturbances [16].

The second most common tumor in our material was fibroma, diagnosed in 13 (21.3%) cases and located in the ventricular septum or ventricular free wall. All children presented various forms of arrhythmia. However, none of our patients had the characteristics of Gorlin syndrome, although according to the literature, about 3–5% of patients with Gorlin syndrome (birthmarks of basal cells) suffer from fibroma [28]. It is known that fibromas are lesions that do not regress, therefore, those tumors are mostly surgically resected. In some cases, heart transplantation is recommended, especially when the tumor is very large and impairs blood flow. In our material, none of the children with a heart tumor had a heart transplant or an implanted pacemaker for arrhythmias. Fibroma, due to its location and shape, always requires differentiation from hypertrophic cardiomyopathy, especially asymmetrical idiopathic hypertrophic subaortic stenosis (IHSS).

In this study, teratoma was in third place with five (8.2%) diagnoses while, in the literature, cardiac teratomas are the second most common primary cardiac tumor in fetuses and neonates [28]. In one child, the tumor was diagnosed prenatally at 28 weeks of pregnancy, which pressed on the right atrium and SVC. The tumor had a tendency towards massive and fast growth and six weeks after delivery, the child died. In the remaining four children, successful surgical excision was the treatment of choice.

The next was myxoma observed only in three (4.9%) patients. Unlike among adults, this type of tumor is rarely diagnosed among children and is found most commonly in the left ventricle. Researchers have defined its global frequency at 7%. Myxoma may also be associated with the Carney Complex [7,29]. According to Careddu et al. [7], the mortality rate among children after the surgical treatment of myxoma is very low, and during a 16-year follow-up, none of the deaths could be directly associated with the tumor. On the other hand, authors describing adult cases recorded different experiences and conclusions. Pacini et al. [30] observed that in-hospital mortality was 3% (3 out of 94 patients) in the myxoma group and this was caused by multifocal embolization, septic shock, and acute renal insufficiency.

In our material, all children had cardiac surgery with good results.

In the case of malignant tumors, the prognosis is dramatically worse. Such tumors appear in 5% of cases and constitute 15–16% of all tumors requiring operational treatment [7,17,18]. The worst prognoses are in the cases of rhabdomyosarcoma and mesothelioma. They manifest with pericardial effusion (even tamponade), arrhythmias, heart failure, or neurological disorders [17]. Unfortunately, these tumors are diagnosed when already in advanced stages with infiltrations and metastasis present [7,19]. Regardless of the type of treatment given, patients rarely survive beyond six months of the initial diagnosis, though the prognosis may be improved by radio- and chemotherapy [3,17,31,32,33]. Recently, clinical trials are underway, which assess the usefulness of temsirolimus in treating patients with rhabdomyosarcoma [34].

In our study, primary malignant heart tumors were highly advanced when they were recognized. We diagnosed two patients with pericardial mesothelioma who died in the early postoperative period due to massive infiltration of the respiratory tract and adjoining tissues. One patient with giant rhabdomyosarcoma in the left atrium also died due to lung and liver metastases. After surgery, the patient was qualified for chemotherapy and radiotherapy. After one year of follow-up, the CT revealed metastases to the left lung and liver, and as a consequence, the patient died.

Basically, the surgery should be performed in heart tumors only in certain cases. In case of benign tumors, surgical treatment should be reserved only for patients with real hemodynamic disorders such as significant blood flow disorder, hypotension, the need for inotropic drugs, or even ECMO treatment. The results of conservative treatment in the group of patients without serious hemodynamic disorders did not significantly differ from those of the cases treated surgically [5]. According to Ying et al. [35], in a group of 16 patients, 15 underwent a complete resection and one a partial one. Surgeries were not performed on malignant tumors. In the presented work, 43.75% of the patients were asymptomatic.

In our study, out of 26 patients (42.7%) that were treated surgically, 17 (65.4%) required urgent surgery. The tumors resected were primarily benign (rhabdomyoma, fibroma, and teratoma) or malignant (mesothelioma and rhabdomyosarcoma). The latter cases had a fatal prognosis, which is consistent with the reports of other authors.

The urgency was justified because there were increasing problems in blood flow as well as heart failure. None of the patients with increasing arrhythmic problems were treated surgically.

In Yin’s material [18], among 131 adult patients with various types of tumors that were treated surgically; 79.4% of the tumors were primary benign heart tumors, while the remaining were metastatic malignant tumors. The author indicated that surgical treatment improved prognoses among patients with operational heart tumors not only due to the tumor’s removal but also due to the reduction in the risk of embolism. It was found that the most common postoperative complication was arrhythmia and 66.41% of the whole group survived over five years. Of those with benign tumors, 82.7% survived five years and more. Of the cases of benign tumors, 4.76% relapsed, while for those with malignant tumors, 31.27%.

In our research, out of 26 surgically treated patients, a majority underwent complete resection. Twenty-two patients (84.6%) showed improvement or stabilization of the tumor, whereas four patients died post-surgery.

### Limitations

This study has several limitations. It is a retrospective study of 15-year material sourced from three centers. During the period considered for this study, diagnostic methods and possible treatments changed significantly. MRI was introduced, though at different times and for different people, in both clinics, as was fetal echocardiography. In a relatively large group of heart and pericardial pediatric tumors, the rate of individual tumor types was low, which makes statistical analysis of significant traits and the demonstration of statistical differences very difficult.

## 5. Conclusions

Pediatric heart tumors do not have specific symptoms and their diagnosis is entirely circumstantial. Diagnosis of a heart tumor in children is not synonymous with fatal prognosis and most of them require only constant observation.

Echocardiography is a basic diagnostic tool; however, MRI and CT currently allow more extensive diagnoses.

Rhabdomyoma is the most common type of tumor among children and its prognosis is rather good. There are no specific manifestations of the tumors, yet a diagnosed arrhythmia needs to be examined to exclude the possibility of a tumor, especially a fibroma. Sclerosis tuberosa symptoms may also indicate a need for further diagnosis in terms of rhabdomyoma.

If multiple heart tumors are found, this can be expected to be a rhabdomyoma.

The surgical removal of tumors should be performed only in cases of increasing blood flow disturbances, and as per a longitudinal observation, it results in an improvement in a majority of cases. Life-saving operation allows improvement, while the prognosis for malignant tumors in children is definitely unfavorable.

## Figures and Tables

**Table 1:** General characteristic of the study group.

	Prenatal Period *n* = 16 (26.2%)	After Birth *n* = 45 (73.8%)	Total *n* = 61
Gender	M-6	M-21	27
	F-10	F-24	34
Age at diagnosis	22–35 weeks	1/365–17.5 year	
Observation time	2–12 weeks	4.3 ± 5.2 years (2 weeks 17,5 years)	
Number of operated patients	0	26 (42.6%)	
Tumor resection	0	total–16 partial–10	26
Diseases involving other organs *n* = 27 (44.2%)			
Tumor in the CNS (ST)	2	14	16
Adrenal hyperplasia		2	2
Bleeding into the CNS		2	2
Pleural effusion	1	1	2
Bilateral hydronephrosis		1	1
Respiratory failure		3	3
Carney’s syndrome		1	1
heart defects coexisting with a heart tumor *n* = 2 (3.3%)			
CoAo		1	1
VSD, ASD II		1	1

Abbreviations: M—male; F—female; CNS—Central Nervous System; ST—Sclerosis Tuberosa; CoAo—Coarctation of the Aorta; VSD—Ventricular Septal Defect; ASD II—Atrial Septal Defect Type II.

**Table 2:** Reasons for cardiologic consultations in children with heart tumors.

**Fetal Period** ***n* = 16 (26.2%)**	
Cardiac symptoms	1 (1.6%)—tamponade
	1 (1.6%)—SVT
	2 (3.3%)—CHD
**After Birth *n* = 45 (73.8%)**	
Neurological diagnosis of ST; Suspected rhabdomyoma	5 (8.2%)
Significant cardiac symptoms (i.e., arrhythmias, fainting, reduced exercise tolerance, organic murmur, tamponade)	30 (49.2%)
Insignificant cardiac symptoms (i.e., innocent murmur)	22 (36.1%)

Abbreviations: SVT—Supraventricular Tachycardia; CHD—Congenital Heart Defect; ST—Sclerosis Tuberosa.

**Table 3:** Detailed analysis of the tumor type, gender, age at diagnosis, frequency, surgical treatment and follow-up.

Type of Tumor	Total (%)	M	F	Mean Age at Diagnosis (month)	Post-Operation Follow-Up			
					Not Operated *n* = 35 (55.3%)		Operated *n* = 26 (42.7%)	
					Mean Time of f-up		Mean Time of f-up	
					4.3 Years		17 Months	
					Improvement/Stable	Death	Improvement/Stable	Death
Rhabdomyoma	37 (60.7)	17	20	6.7	27	1	8	1
Fibroma	13 (21.3)	5	8	13.2	5	1	7	
Teratoma	5 (8.2)	1	4	1.7		1	4	
Myxoma	3 (4.9)	2	1	124			3	
Mesothelioma	2 (3.3)	1	1	167				2
Rhabdomyosarcoma *	1 (1.6)	1	0	192				1
Total(%)	61 (100.0)	27	34	84.1	32 (91.4)	3 (8.6)	22 (84.6)	4 (15.4)

Abbreviations M—male; F—female. * In this group was a 16-year-old boy-athlete who played football for 10 years. The sports doctor referred him for cardiological consultation because the standard planned stress test found worse exercise tolerance (less metabolic equivalents (METs)) but without specific changes in the record. During the cardiological consultation, a giant tumor in the left atrium was detected, significantly hindering the flow of blood to the left ventricle through the mitral valve (Figure 1). The cardiac surgery was performed in an emergency, and only subtotal resection was performed because of left atrial wall infiltration (Figure 2). Histopathological diagnosis was rhabdomyosarcoma. Despite the satisfactory early results after surgery, chemotherapy, and radiotherapy, the patient died of lung and liver metastases.

**Table 4:** Location of tumors.

Location	Number (%)		Number
Ventricle	30 (49.1)	left	11
		right	8
		both	11
Interventricular Septum	9 (14.9)		
Atria of the Heart	6 (9.8)	left	3
		right	3
Pericardium	8 (13.1)	teratoma mesothelioma rhabdomyosarcoma	5 2 1
Various Locations	8 (13.1)		
Total	61 (100,0)		

**Table 5:** Number of tumors in the heart and sclerosis tuberosa.

Number	Total *n* = 61 (100%)	ST *n* = 19 (100%)
Single	43 (70.5)	4 (9.3)
2–3	7 (11.5)	5 (71.4)
>3	11 (18.0)	10 (90.9)

Abbreviations: ST-sclerosis tuberosa.

**Table 6:** Heart tumors that led to the deaths of children.

	Age	Localization	Operation	Diagnosis Hist-Path	f-up	Death	Clinical Course
Prenatal							
1	20 weeks	LV, RV	no	rhabdomyoma	4 weeks	yes	massive progression
2	28 weeks	impression on RA and SVC	no	teratoma	6 weeks	yes	massive progression
Newborn							
3	1 day	LA, RVOTO	yes	rhabdomyoma	7/days	yes	massive progression
4	3 weeks	IVS, RV, LV	no	fibroma	14 years	yes	massive progression, VT
Adolescent							
5	10 years	pericardium	yes	mesothelioma	2 years	yes	tamponade, massive metastases to mediastinum
6	17 years	pericardium	yes	mesothelioma	2 months	yes	tamponade, massive metastases to mediastinum
7	16 years	LA	yes	rhabdomyosarcoama	1 year	yes	metastases to lung and liver

Abbreviations: LV—Left Ventricle; RV—Right Ventricle; SVC—Superior Vena Cava; RA—Right Atrium; LA—Left Atrium; IVS—Interventricular Septum; RVOTO—Right Ventricular Outflow Tract Obstruction; VT—Ventricular Tachycardia.
